# Adaptive Temporal-Aware Feature Selection for Driver Fatigue Detection

**DOI:** 10.3390/s26216758

**Published:** 2026-10-26

**Authors:** Runze Zhu, Guodong Zha, Xiaoqian Cao, Jie Fang

**Affiliations:** 1 School of Telecommunication and Information Engineering, Xi’an University of Posts and Telecommunications, Xi’an 710121, China; 2 School of Electrical and Control Engineering, Shaanxi University of Science and Technology, Xi’an 710021, China; 3 School of Artificial Intelligence, Optics and Electronics (iOPEN), Northwestern Polytechnical University, Xi’an 710072, China

**Keywords:** driver fatigue detection, temporal perception, efficient inference, feature selection

## Abstract

Achieving favorable compatibility between detection performance and inference efficiency has long been a core bottleneck restricting the practical deployment of vision-based driver fatigue detection algorithms on vehicle-mounted embedded devices. In this work, we propose an adaptive temporal-aware feature selection paradigm for driver fatigue detection to address temporal isolation and computational redundancy problems. Specifically, we propose a temporal dual-stream fusion (TDF) module to generate unified embeddings of highly discriminative video features, laying a solid feature foundation for superior final performance. Additionally, we design an adaptive feature selection (AFS) mechanism that evaluates the necessity of executing each stream based on confidence estimation, eliminating redundant computations with acceptable slight accuracy loss. In addition, the experimental results on the challenging NTHU-DDD dataset demonstrate the superiority of our method, especially from the perspective of balancing performance and efficiency.

## 1. Introduction

Road traffic safety remains a critical social issue worldwide [1]. Statistics indicate that driver fatigue is responsible for roughly 12%–20% of all road accidents and over 40% of fatal crashes, with a mortality rate far exceeding ordinary traffic incidents. Long-duration driving, monotonous road conditions and insufficient sleep all trigger mental and physical fatigue, which impairs drivers’ reaction speed, visual perception and decision-making capacity, drastically raising collision risks. As intelligent driving and driver monitoring systems (DMSs) gain widespread deployment, non-contact vision-based fatigue detection has emerged as a research hotspot thanks to its low hardware cost, high user acceptance and convenient deployment without invasive sensors.

Existing vision-based fatigue detection paradigms can be divided into single-stream spatial models and naive spatio-temporal dual-stream architectures. Single-stream CNNs merely extract static facial cues such as eye openness, mouth yawns and head poses from isolated frames, ignoring the continuous dynamic evolution of fatigue symptoms over time, thus suffering severe performance degradation under complex interference including drastic day–night illumination shifts and partial occlusion by regular or reflective sunglasses. Conventional dual-stream networks separately process spatial appearance and temporal motion information to capture fatigue-related dynamic patterns, yet they adopt fixed parallel inference for all video frames without screening redundant feature branches. This rigid computation pattern generates massive useless feature calculations and restricts the model’s real-time inference speed, failing to strike a balance between detection accuracy and computational efficiency on embedded vehicle-end devices.

**Figure 1:**
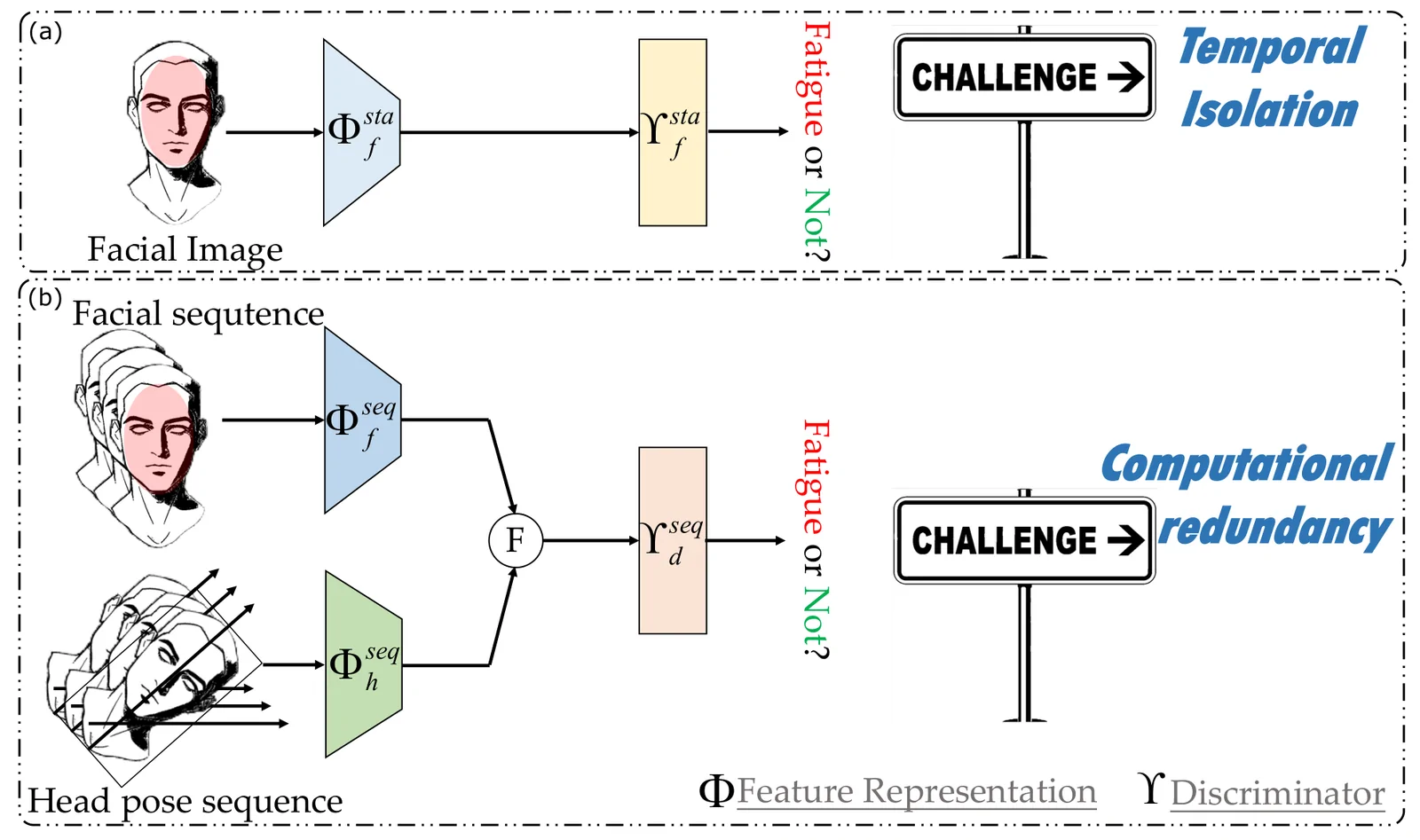
Challenges of existing methods in driver fatigue detection: (**a**) temporal isolation in single-frame facial-image processing; (**b**) computational redundancy in fixed dual-stream processing.

To address the two major challenges of temporal isolation and computational redundancy, as shown in Figure 1, we design a unified driver fatigue detection paradigm based on adaptive temporal-aware feature selection. This paradigm centers on two points, including temporal dual-stream fusion (TDF) and adaptive feature selection (AFS). The former focuses on temporal perception, while the latter takes efficient inference into consideration. Specifically, we introduce a Gated Recurrent Unit (GRU) to construct a temporal feature fusion network. By capturing long-term dependencies across consecutive frames and learning the evolution patterns of hidden states, the network generates dynamic frame-level weights, significantly enhancing the ability of the model to capture the evolutionary process of fatigue behaviors and its robustness against interference. In addition, we utilize a lightweight regression network to estimate the confidences of different branches to dynamically select the optimal inference path accordingly, thereby achieving on-demand allocation of computational resources.

Under the premise of maintaining high-precision detection for critical fatigue samples, this chapter eliminates unnecessary computation during prolonged wakeful states, achieving synergistic optimization of temporal feature mining and inference efficiency at the algorithm architecture level, providing a novel and reliable paradigm for energy-efficient fatigue detection in complex scenarios. The main contributions of this paper are summarized as follows:

- We propose a temporal dual-stream fusion (TDF) module to generate unified embeddings of highly discriminative video features, laying a solid feature foundation for superior final performance.
- We design an adaptive feature selection (AFS) mechanism that evaluates the necessity of executing each stream based on confidence estimation, eliminating redundant computations and performance degradation.
- We integrate our proposed TDF and AFS modules into a unified framework for driver fatigue detection, which simultaneously maintains favorable detection accuracy and high inference speed.
- We conduct extensive experiments on the NTHU-DDD dataset, and the results show significant advantages of our method, especially from the perspective of balancing performance and efficiency.

The rest of this paper is organized as follows. Section 2 reviews the existing methods for detecting driver fatigue and multi-source fusion in brief. Section 3 details the proposed driver fatigue detection algorithm based on adaptive temporal-aware feature fusion. Section 4 reports and analyzes the experiments. Section 5 concludes this paper.

## 2. Related Works

### 2.1. Driver Fatigue Detection

Driver fatigue has been identified as one of the leading causes of traffic accidents worldwide, accounting for approximately 15% to 30% of all road accidents [2,3]. Accurate and timely detection of driver fatigue is therefore essential for improving road safety. According to the types of signals employed, existing fatigue detection methods can be broadly categorized into three classes [2].

The first category relies on physiological signals, including electroencephalography (EEG), electrocardiography (ECG), electrooculography (EOG), and electromyography (EMG), which directly reflect the driver’s internal physiological state [4,5]. Among these, EEG-based fatigue detection is considered to be one of the more reliable approaches due to its high temporal resolution and its ability to directly reflect cortical electrical activity [6,7]. Recent studies have demonstrated that effective classification of multi-level fatigue states can be achieved through power spectral density (PSD) feature extraction combined with lightweight convolutional neural networks [7]. Furthermore, cross-modal methods based on the fusion of EEG and EOG signals have shown good stability and generalization ability in cross-subject detection [8]. Although physiological-signal-based methods achieve high accuracy, they typically require the driver to wear contact sensors, which may cause discomfort during prolonged driving. The relatively high cost of equipment also limits their large-scale deployment in real-world scenarios [4].

The second category relies on vehicle driving data, such as steering wheel angle, lane departure, and vehicle speed, which indirectly infer fatigue through the degradation of driving performance [9]. In recent years, with the popularization of infrastructure such as the ETC gantry system, methods based on vehicle operation situation analysis have been gradually adopted for auxiliary fatigue driving judgment [10]. However, these approaches are susceptible to individual driving habits and road conditions. The significant variation in operating styles among different drivers limits the generalization ability of such models [9,10].

The third category, which has gained significant attention in recent years, employs vision-based techniques using in-vehicle cameras to capture facial expressions, eye states, mouth movements, and head poses for fatigue recognition [11,12,13]. The advantages of this approach lie in its non-invasive nature and low hardware cost. With the advancement of deep learning, particularly convolutional neural networks and object detection algorithms, vision-based methods have become the mainstream direction of current research [11,14,15]. In terms of specific implementations, researchers have developed various facial-feature-based fatigue recognition systems that comprehensively determine fatigue status by detecting indicators such as PERCLOS, yawn frequency, and head posture changes [11,12]. Zhang et al. proposed lightweight and real-time improvements to the YOLOv8n model, making it more suitable for deployment on in-vehicle embedded devices [15]. In addition, the combination of facial features and body movements has also been applied to driving risk recognition in complex scenarios [16].

Despite these advances, existing vision-based methods still face two major challenges. First, most approaches rely on single-frame images for fatigue judgment, neglecting the temporal continuity and dynamic cumulative effects of fatigue behaviors, which develop gradually over time [17]. Second, the computational demands of deep neural networks pose challenges for deployment on resource-constrained in-vehicle embedded platforms, requiring a trade-off between model accuracy and inference speed [15,17].

Dynamic inference methods aim to adaptively allocate computational resources according to input complexity, which is promising for addressing this dilemma. Representative works adopt adaptive routing or conditional computation to activate partial network branches for different samples [18,19]. Meanwhile, several temporal models have been developed based on the NTHU-DDD dataset for video-based driver distraction detection [20]. However, these existing temporal models adopt fixed computation graphs for sequential frames and cannot dynamically adjust computation overhead. Different from these, our AFS module implements input-aware adaptive routing. It dynamically selects multi-modal temporal features and allocates computation resources on demand, which provides a flexible performance–efficiency trade-off for video-based distraction recognition.

### 2.2. Multi-Source Fusion

To overcome the limitations of single-modality approaches, multi-source fusion has emerged as an important approach to enhancing the robustness of fatigue detection systems [21,22]. The core rationale is that different data sources provide complementary information: visual features capture observable facial behaviors and postural changes, physiological signals reflect the driver’s internal state variations, and vehicle dynamics data indicate the degradation of driving performance [21]. By integrating these complementary modalities, the system can maintain reliable detection performance even when a single sensor fails or partial information is missing [22].

#### 2.2.1. Multi-Source Data Integration

Multi-source data integration aims to organize and preprocess heterogeneous data from different sensors, providing a consistent data foundation for subsequent fusion [8,21]. In driver fatigue detection, common data sources include visual sensors (cameras), physiological sensors (EEG/ECG/EOG), and vehicle CAN bus data [8,21]. These sources differ significantly in sampling frequency, data format, and physical meaning. For example, visual data typically has a high spatial resolution but a low frame rate, while physiological signals are time-series data with high sampling rates. How to achieve effective spatiotemporal alignment is the primary challenge.

#### 2.2.2. Multi-Source Feature Fusion

Feature fusion is the core component of multi-source integration systems, aiming to combine features extracted from different modalities into a more discriminative unified representation [22,23]. According to the stage at which fusion occurs, it can be divided into early fusion (integrating raw data before feature extraction), late fusion (combining decision results after independent processing of each modality), and hybrid fusion (balancing both approaches) [22]. In recent years, attention mechanisms have been widely applied to multi-modal feature fusion, significantly improving fusion performance by adaptively learning the importance weights of different modal features [21,22,23]. In addition, Transformer-based fusion methods have also demonstrated good performance in multi-modal fatigue detection [8,21].

#### 2.2.3. Multi-Source Collaborative Decision-Making

At the highest level of the fusion hierarchy, collaborative decision-making integrates discrimination results from multiple modalities to make the final fatigue state judgment [22,23]. Common decision-level fusion methods include voting, weighted averaging, and Bayesian inference [22]. In practical deployment, collaborative decision-making must balance detection accuracy and computational efficiency, especially in in-vehicle embedded environments [15,22]. Related studies have pointed out that fatigue detection systems for real-world driving scenarios need to further focus on personalized modeling and decision interpretability to accommodate individual differences in fatigue expression among different drivers [23,24].

## 3. Proposed Method

**Figure 2:**
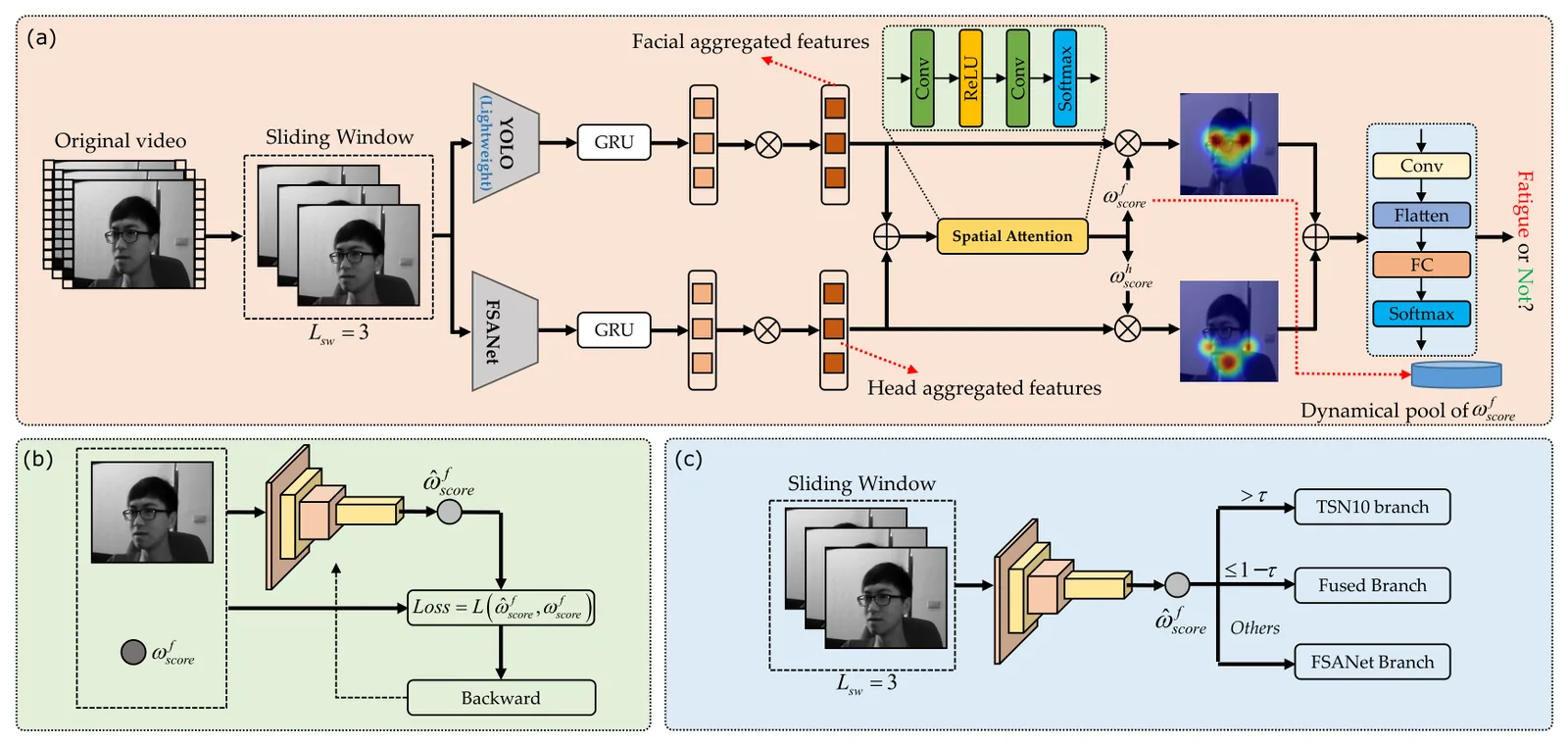
Framework of the temporal-aware adaptive feature selection algorithm: (**a**) overall temporal dual-stream fusion network; (**b**) training process of the adaptive feature selection regressor; (**c**) adaptive branch selection during inference. The background colors distinguish the three components, black arrows indicate the processing flow, and red dotted arrows highlight feature aggregation and score-pool updates.

The adaptive feature selection algorithm with temporal information mainly includes two core stages: temporal dual-stream fusion and adaptive feature selection, as shown in Figure 2. In the first stage of temporal dual-stream fusion, the model receives raw video frames and builds a sliding window of size $L_{sw}$. Video frames are fed into the facial branch and the head branch separately. Each branch adopts a GRU to model temporal dependencies over continuously extracted spatial features and captures long-range correlations among adjacent frames. The two groups of features then enter a lightweight attention fusion module, which adaptively calculates fusion weights for each feature stream. Weight-fused joint features go to a classifier for fatigue-state prediction. Meanwhile, this stage extracts fusion weights from the facial branch. These weights act as supervision signals to train the lightweight regression network in the second stage.

In the second stage of adaptive feature stream selection, the lightweight regression network takes pseudo-labels generated in the first stage as supervision signals for parameter optimization. The network updates its weights via loss calculation and back-propagation. It thereby learns to assess the importance of different feature branches from a single frame. During inference, the regression network accepts target frames sampled from the sliding window. It outputs the facial-branch prediction score ${\hat{\omega }}_{\mathrm{score}}^{f}$ at low computational cost. The system applies dynamic branch selection by comparing this score with a predefined threshold τ. This architecture cuts redundant computation while preserving detection accuracy. It achieves a good trade-off between accuracy and real-time performance for embedded vehicle platforms.

### 3.1. Temporal Dual-Stream Fusion

This section analyzes the theoretical basis, network design and implementation details of each module in the first stage. It explains how the model produces discriminative unified embeddings of temporal dual-stream fatigue features from video sequences.

#### 3.1.1. Temporal Sliding Window

Fatigue behaviors show strong temporal continuity. Blinking, yawning and head drooping develop across successive frames. Single-frame inputs miss important dynamic variations. Nevertheless, 3D-CNN or LSTM [25] bring heavy computation and memory burdens, which harm real-time inference in practical deployment. This work introduces a temporal sliding-window module. It gathers sufficient temporal context without sacrificing computational efficiency. The module constructs local temporal segments using historical frames plus the current frame during data loading. Only past frames serve as context; no future-frame data is involved. Consequently, the model infers purely from observed history and satisfies causality requirements for real-time systems.

(1) Window Structure Constraints

The system imposes hard constraints for sliding-window initialization to guarantee valid sampling. Let $s_{w}$ denote the total window size and $s_{f}$ denote the number of preceding historical frames. Future frames are strictly excluded, so the count of subsequent frames equals zero. The window size must follow the causal constraint in Equation (1).

(1) $$s_{w}=s_{f}+1$$

Here, +1 accounts for the target frame itself. This constraint guarantees complete temporal context. The temporal window slides over video frames with a fixed stride s=1. Every frame becomes a target frame with its own temporal context. This sampling strategy enables the model to capture dynamic changes among neighboring frames.

(2) Boundary Constraint Addressing Strategy

Consider a video sequence with *L* total frames and index set I={0,1,…,L−1}. For a target frame indexed by i∈I, the sliding-window theoretically yields the context index set in Equation (2).

(2) $$T_{i}=\left\{t|t=i+\delta ,\;\delta \in \left[-s_{f},0\right]\right\}$$

Indices t<0 may appear when *i* lies near the video start. Conventional zero-padding introduces severe artificial abrupt noise at sequence boundaries and breaks the continuous temporal semantics of fatigue motions. To mitigate boundary artifacts, this module adopts a static-continuation strategy. The underlying assumption states that driver actions remain unchanged for a short period right before recording starts. A mapping function Φ(t) remaps all out-of-bound historical indices to the first valid frame, as given in Equation (3).

(3) $$\Phi \left(t\right)=\left\{\begin{array}{cc} 0, & t<0\\ t, & t\geq 0 \end{array}\right.$$

Applying Φ(t) produces the practical sampling index set $P_{i}=\left\{\Phi \left(t\right)|t\in T_{i}\right\}$. The sliding window repeatedly samples the initial-frame features at video beginnings. Besides avoiding leakage of future information, this setup suppresses artificial high-frequency noise. It generates well-behaved input streams that comply with physical priors for subsequent feature extraction networks.

#### 3.1.2. Temporal Weight Generation

**Figure 3:**
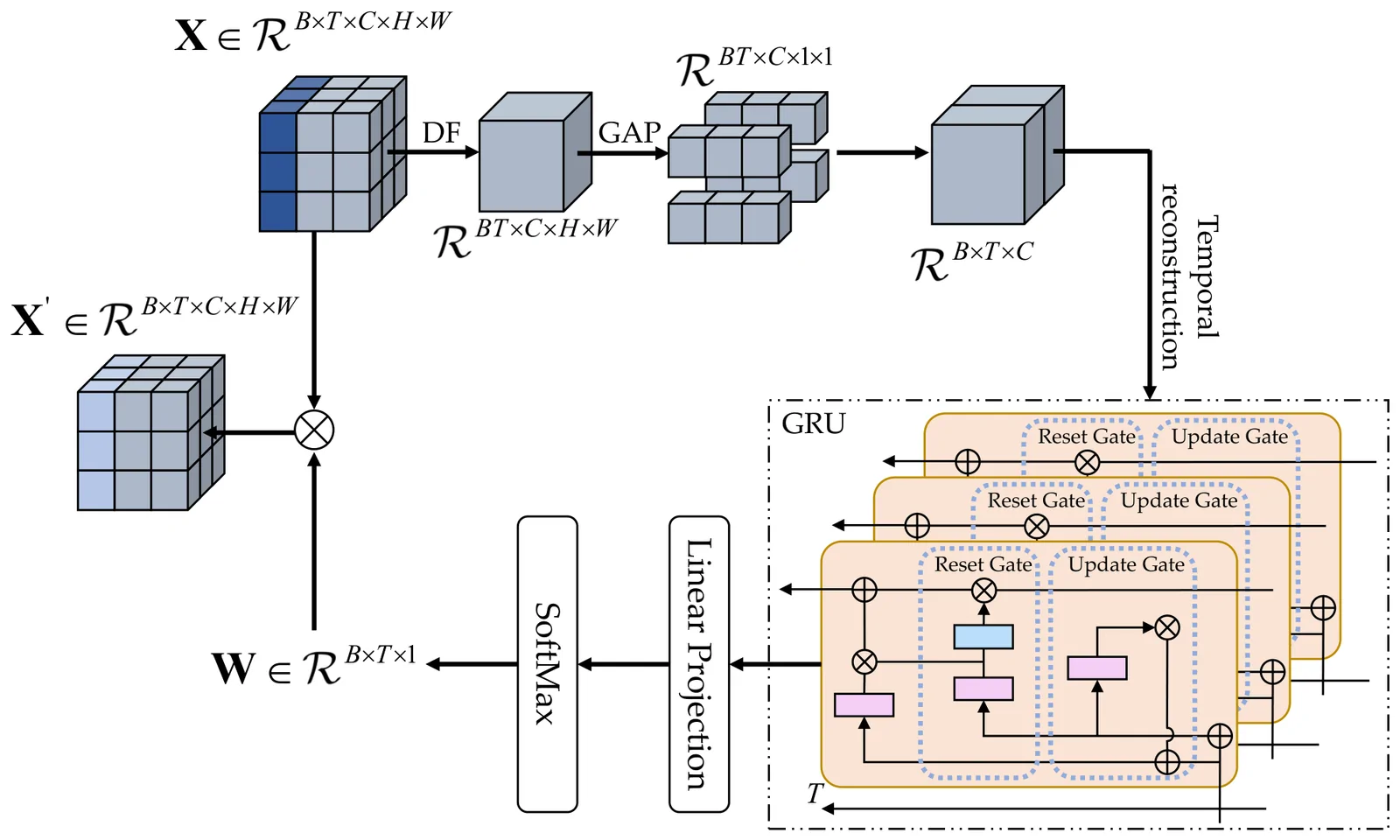
Gated recurrent unit weight generation module.

We build GRU-based temporal weight generators for both facial and head-pose branches. These generators measure frame-wise contributions within each sliding window, as illustrated in Figure 3. The module captures temporal dependencies across frames and produces dynamic per-frame weights from evolving hidden states. Each branch computes weighted sums over multi-frame window features using these temporal weights. Discrete temporal sequences aggregate into robust context-aware feature representations.

(1) Spatial Dimensionality Reduction and Temporal Sequence Constructiony

We first merge batch and temporal dimensions of tensor $X\in R^{B\times T\times C\times H\times W}$ and reshape it into $R^{(B\cdot T)\times C\times H\times W}$. Two-dimensional adaptive GAP compresses spatial resolution to 1×1. Reduced features are reshaped back into temporal-sequence tensor $V\in R^{B\times T\times C}$ as input for the gating network.

(2) Temporal Modeling Based on GRU

Tensor V feeds into the GRU unit. Its internal gating mechanism captures deep temporal correlations. For input $v_{t}$ at time t∈[1,T], GRU updates its hidden state following Equation (4).

(4) $$h_{t}=\left(1-z_{t}\right)⊙h_{t-1}+z_{t}⊙{\tilde{h}}_{t}$$

where $z_{t}$ is the update gate, which is defined in Equation (5).

(5) $$z_{t}=\sigma \left(W_{z}\cdot \left[h_{t-1},\;v_{t}\right]+b_{z}\right)$$

Variable ${\tilde{h}}_{t}$ in Equation (4) corresponds to the candidate hidden state in Equation (6).

(6) $${\tilde{h}}_{t}=\tanh\left(W_{h}\cdot \left[r_{t}⊙h_{t-1},\;v_{t}\right]+b_{h}\right)$$

where $r_{t}$ denotes the reset gate from Equation (7).

(7) $$r_{t}=\sigma \left(W_{r}\cdot \left[h_{t-1},\;v_{t}\right]+b_{r}\right)$$

The output hidden-state sequence $H=\{h_{1},h_{2},\ldots ,h_{T}\}$ encodes full contextual memory inside each sliding window.

(3) Attention Mapping and Feature Modulation

Hidden states H project toward scalar space to produce per-frame weights. A linear layer extracts temporal features. A softmax operator in Equation (8) normalizes weights over temporal dimension *T*.

(8) $$w_{t}=\frac{\exp\left(\phi (h_{t})\right)}{\sum_{k=1}^{T}\exp\left(\phi (h_{k})\right)},\;t=1,2,\ldots ,T$$

where ϕ(·) stands for fully connected linear mapping. Weight tensor $w=\left\{w_{1},w_{2},\ldots ,w_{T}\right\}\in R^{T\times d}$ modulates raw input X via broadcast element-wise multiplication from Equation (9) and yields weighted features $\tilde{X}$.

(9) $$\tilde{X}=w⊙X$$

#### 3.1.3. Dual-Stream Attention Fusion

**Figure 4:**
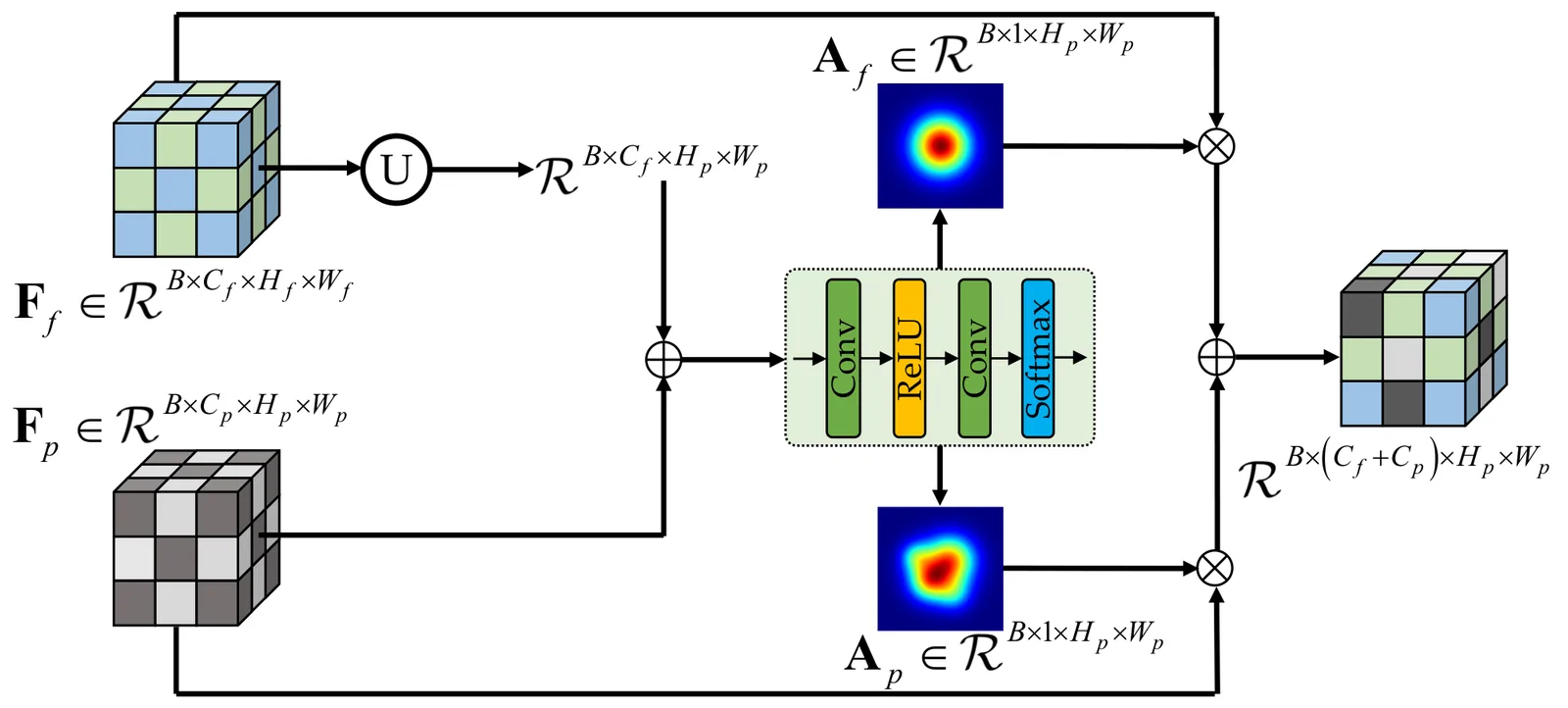
The inner architecture of the Dual-Stream Attention Fusion Module.

Features from two branches go through independent preprocessing. This paper proposes a dual-stream attention fusion module (DSAF), shown in Figure 4. It adopts a cascade pipeline including scale alignment, nonlinear purification and feature modulation. The module aggregates information efficiently within a unified feature space.

(1) Spatial Scale Alignment and Joint Representation

Denote facial features from the YOLO branch as $F_{f}\in R^{B\times C_{f}\times H_{f}\times W_{f}}$ and pose features from FSANet [26] as $F_{p}\in R^{B\times C_{p}\times H_{p}\times W_{p}}$. To resolve spatial-resolution mismatch, bilinear upsampling aligns $F_{f}$ to the spatial grid of pose features. The module concatenates features along the channel dimension and builds joint tensor $F_{\mathrm{cat}}\in R^{B\times \left(C_{f}+C_{p}\right)\times H_{p}\times W_{p}}$ with complete semantic information.

(2) Attention Purification

Simple concatenation introduces redundancy. DSAF builds a lightweight attention network to capture complex nonlinear correlations among features. First, 1×1 point-wise convolution reduces joint-feature dimensions. A ReLU activation enhances nonlinear representation capacity. A second point-wise convolution maps features onto 2D channel planes.

(3) Deep Feature Modulation

Once spatial attention maps are obtained, DSAF refines weights for each feature-map coordinate using the Hadamard product. It completes deep feature modulation as formulated in Equation (10).

(10) $$F_{\mathrm{out}}=\left[F_{f}^{\mathrm{up}}⊙A_{f};\;F_{p}⊙A_{p}\right]\in R^{B\times \left(C_{f}+C_{p}\right)\times H_{p}\times W_{p}}$$

This attention mechanism acts as a spatial filter. It strengthens high-confidence target regions and suppresses background noise caused by spatial alignment. It outputs robust temporal dual-stream fused features.

### 3.2. Adaptive Feature Stream Selection

The first-stage temporal dual-stream fusion model delivers competitive performance and generalization for fatigue-driving recognition. However, inference requires full activation of both feature branches plus the fusion module, which brings high computational cost. Different driving scenarios rely unequally on each branch. When discriminative cues concentrate heavily within one branch (for instance, obvious facial fatigue or abnormal head pose), running the other whole branch produces redundant computation and slows system responses. We therefore design a condition-triggered inference mechanism. The model selects computation paths adaptively according to sample-wise feature distributions and achieves on-demand computation.

Based on the Stage-I model, we propose an adaptive feature-selection strategy. A lightweight regression network fits branch-importance distributions learned by the backbone model. During inference, regression-network predictions determine the actual execution path. The original static full dual-stream network transforms into an adaptive inference architecture, and overall inference efficiency improves.

#### 3.2.1. Label Construction

When the Stage-I backbone model converges, its internal dual-stream fusion module outputs branch-level fusion weights for each training sample: $\omega_{\mathrm{score}}^{f}$ for the facial branch and $\omega_{\mathrm{score}}^{h}$ for the head-pose branch. These weights satisfy normalization $\omega_{\mathrm{score}}^{f}+\omega_{\mathrm{score}}^{h}=1$. Predicting both values creates dimensional redundancy. We extract only facial-branch weight $\omega_{\mathrm{score}}^{f}$ as pseudo-labels for regression-network supervision, as defined in Equation (11).

(11) $$\omega_{\mathrm{score}}^{f}=\mathrm{GAP}\left(A_{f}\right)$$

#### 3.2.2. Network Design

**Figure 5:**
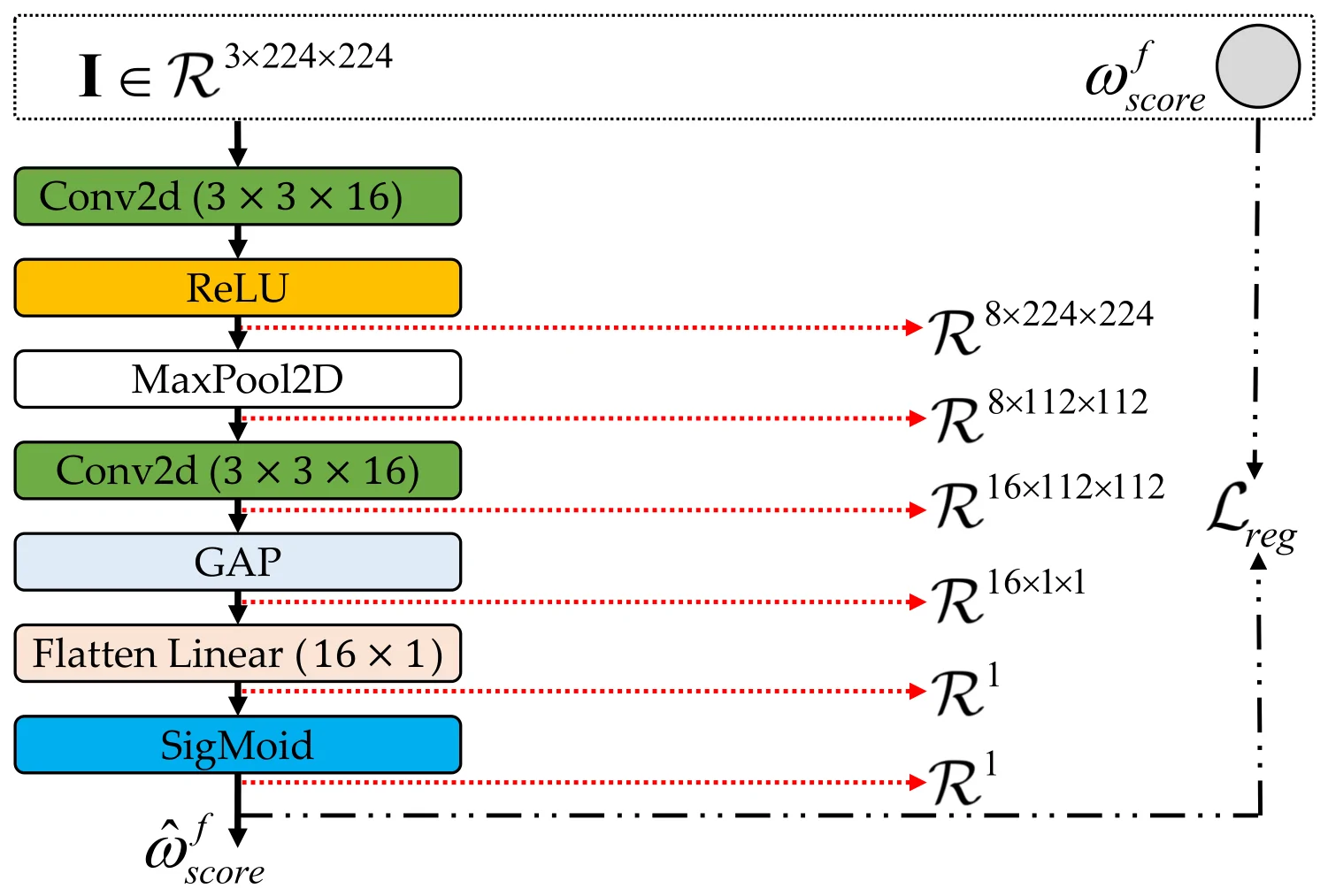
Architecture of the regression network model. Colored blocks distinguish different network operations, black arrows indicate the forward data flow, red dotted arrows indicate the dimensions of intermediate feature representations, and the black dash-dotted line represents the loss-related connection between the predicted and target scores.

This subsection describes the lightweight regression network serving as the front-end decision module. To satisfy strict low-latency requirements for real-time inference, the network takes only the current-frame $X_{t}\in R^{3\times 224\times 224}$ within the sliding window as input for both training and inference. This design removes computation overhead for sequential video processing. Figure 5 shows its overall structure. Input $X_{t}$ passes through convolution layers, max-pooling layers and adaptive average pooling for spatial downsampling. Features flatten and feed into fully connected layers. A final Sigmoid guarantees probability-like weight outputs. Forward propagation yields predicted one-dimensional branch weight ${\hat{\omega }}_{\mathrm{score}}^{f}$ in Equation (12).

(12) $${\hat{\omega }}_{\mathrm{score},t}^{f}=\mathrm{Sigmoid}\left(F_{\theta }\left(X_{t}\right)\right)$$

where $F_{\theta }$ denotes the regression network with parameters θ.

#### 3.2.3. Network Optimization

This module trains with MSE loss $L_{\mathrm{reg}}$. It drives predicted weights to approximate pseudo-labels dynamically generated by the primary model. Given a mini-batch with *N* samples, the loss function follows Equation (13).

(13) $$L_{\mathrm{reg}}=\frac{1}{N}\sum_{i=1}^{N}{\left(\omega_{\mathrm{score},i}^{f}-{\hat{\omega }}_{\mathrm{score},i}^{f}\right)}^{2}$$

Back-propagation minimizes $L_{\mathrm{reg}}$ and updates regression-network parameters θ. Complex decision knowledge transfers toward this lightweight network.

#### 3.2.4. Branch Selection

For practical inference, the system introduces confidence threshold 0.5<τ<1.0. It builds a three-way decision function s(X) purely from branch weight ${\hat{\omega }}_{\mathrm{score}}^{f}$, as given in Equation (14).

(14) $$s\left(X\right)=\left\{\begin{array}{cc} \psi_{\mathrm{face}}, & {\hat{\omega }}_{\mathrm{score}}^{f}\geq \tau \\ \psi_{\mathrm{head}}, & {\hat{\omega }}_{\mathrm{score}}^{f}\leq 1-\tau \\ \psi_{\mathrm{fuse}}, & 1-\tau <{\hat{\omega }}_{\mathrm{score}}^{f}<\tau \end{array}\right.$$

The decision rules work as follows. If one-branch prediction exceeds threshold τ, information from that single branch suffices. The system activates only the corresponding single-branch network ($\psi_{\mathrm{face}}$ or $\psi_{\mathrm{head}}$). When neither branch meets the threshold, single-branch signals become insufficient. The system conservatively enables dual-branch fusion network $\psi_{\mathrm{fuse}}$. The mechanism adds negligible overhead at the front end and adaptively optimizes computational-resource allocation.

#### 3.2.5. Supplementaries

The decision logic maps feature saliency to computational-resource assignment. It supports three execution modes.

- Face-dominant mode ($\psi_{\mathrm{face}}$): When ${\hat{\omega }}_{\mathrm{score}}^{f}\geq \tau$, facial fatigue signs such as frequent yawning or eye closure dominate within the current frame. The decider enables only the facial perception branch and skips unnecessary head-pose processing.
- Head-dominant mode ($\psi_{\mathrm{head}}$): When ${\hat{\omega }}_{\mathrm{score}}^{h}\geq \tau$ (${\hat{\omega }}_{\mathrm{score}}^{f}\leq 1-\tau$), drowsiness induces strong head motions such as nodding. Head-pose features dominate. The decider considers single-branch information adequate and activates only the head-pose branch. Computation for the facial branch is skipped.
- Dual-stream fusion mode ($\psi_{\mathrm{fuse}}$): When predictions fall within (1−τ,τ), neither feature stream provides enough discriminative power. A typical case includes slightly closed eyes together with mild head tilting. The decider activates dual-branch fusion. Rich spatiotemporal features preserve recognition accuracy.

#### 3.2.6. Hyperparameter τ

Threshold τ acts as a key hyperparameter balancing inference accuracy and computational efficiency. When τ approaches 1.0, the criteria become strict. The system mostly falls back to full dual-branch fusion $\psi_{\mathrm{fuse}}$. This setting yields upper-bound classification accuracy yet brings maximum computation cost. When τ gets close to 0.5, thresholds relax. Single-branch inference triggers frequently. Latency reduces at the risk of misclassification. We calibrate τ on validation sets. The model can adapt flexibly to different deployment hardware constraints.

## 4. Experiments

### 4.1. Dataset

**Figure 6:**
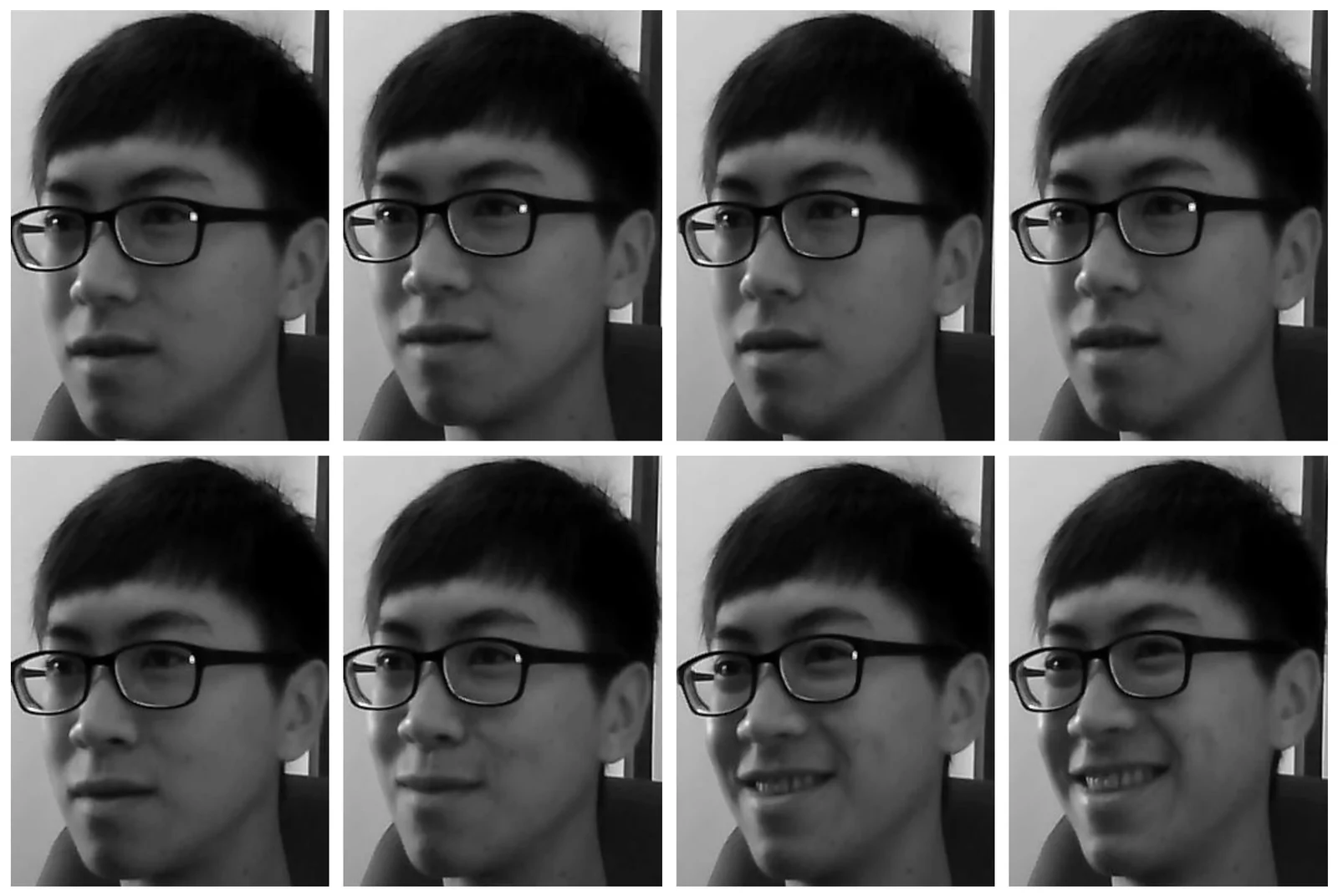
Some examples from the NTHU-DDD dataset.

To overcome the limitation that single static frames struggle to capture continuous fatigue variations, this paper focuses on temporal dynamic modeling instead of static spatial feature extraction. We adopt the NTHU Driver Drowsiness Detection video dataset (NTHU-DDD)  [20] as the primary evaluation benchmark, with sample illustrations visualized in Figure 6. The dataset contains 36 high-definition infrared and visible video sequences collected in simulated driving scenarios. It covers diverse challenging conditions, including drastic day--night light changes and partial occlusion by sunglasses, and it includes various fatigue behaviors such as yawning and frequent head lowering. The fine-grained frame-wise binary annotations and multi-dimensional facial/head pose attributes support reliable evaluation of dynamic fatigue detection.

To avoid data leakage and ensure rigorous evaluation, we implement a strict subject-independent partition protocol.

All videos from a single subject are entirely assigned to one subset (training, validation, or test), with no cross-subset subject overlap. We abandon stride-1 overlapping sampling to prevent highly similar frames from spreading across different splits. The dataset is divided at an approximate ratio of 70%:15%:15% by subject identity. Specifically, among the total 36 subjects, 25 subjects are used for training, 5 for validation and 6 for testing. The validation set is used exclusively for hyperparameter tuning, while the test set is only utilized for final evaluation and never involved in parameter optimization.

Overall, the NTHU-DDD dataset provides comprehensive temporal and scene diversity to validate our temporal feature extraction framework, which complements static spatial modeling and forms a complete experimental pipeline for dynamic driver fatigue detection.

### 4.2. Evaluation Metrics

Following most existing works based on the NTHU-DDD benchmark, overall accuracy is adopted as the major evaluation metric for performance comparison.

### 4.3. Implementation Details

All experiments are implemented based on PyTorch 2.4.1. The input frame size is set to 224×224. We use the SGD optimizer, with the initial learning rate set to $1\times 10^{-4}$.

To obtain statistically stable results and reduce random initialization bias, all main experiments are repeated with five different random seeds. We report mean performance together with standard deviation (mean±σ) for quantitative comparison. Hyper-parameters are determined solely based on the validation set, and no test-set feedback is used during parameter searching. In addition, The face backbone is pre-trained on WIDER-Face [27]. We expand detected face boxes by 15% for face region cropping, and we crop local regions via human keypoints. The model is trained end-to-end for 100 epochs with a cosine learning rate decay.

### 4.4. Experimental Setup

To ensure the fairness of model evaluation and the rigor of comparative experiments, this work adopted the same basic hardware and software experimental environment for different algorithms. The specific hardware configuration consisted of four NVIDIA TITAN XP graphics processing units (GPUs). The software runtime environment included Linux, CUDA 11.8, Python 3.8, and PyTorch 2.4.1.

Regarding the basic training parameters of the model, the core hyperparameters of the facial feature extraction, head pose estimation, and dual-stream feature fusion networks are shown in Table 1.

To evaluate the results of different methods, we utilized Accuracy (Acc.), Precision (P), Recall (R), F_1_-score (F_1_), Frames per Second (FPS), and Number of Parameters (Para.) as the core evaluation criteria to measure their performance. The definition of Acc. is formulated in Equation (15).

(15) $$\mathrm{Acc}.=\frac{\mathrm{TP}+\mathrm{TN}}{\mathrm{TP}+\mathrm{TN}+\mathrm{FP}+\mathrm{FN}}\times 100\%$$

where TP, TN, FP, and FN denote the numbers of true positives, true negatives, false positives, and false negatives, respectively. The definitions of precision (P) and recall (R) are given in Equation (16).

(16) $$\begin{array}{cc} P=\frac{\mathrm{TP}}{\mathrm{TP}+\mathrm{FP}}\times 100\%, & R=\frac{\mathrm{TP}}{\mathrm{TP}+\mathrm{FN}}\times 100\% \end{array}$$

The F_1_-score is the harmonic mean of P and R and is defined in Equation (17).

(17) $$F_{1}=\frac{2\cdot P\cdot R}{P+R}$$

FPS denotes the number of frames processed per second and is calculated using Equation (18).

(18) $$\mathrm{FPS}=\frac{N_{\mathrm{total}}}{T_{\mathrm{total}}}$$

where $N_{\mathrm{total}}$ is the total number of frames in the test samples, and $T_{\mathrm{total}}$ is the total time required to process these samples. Para. denotes the number of parameters in the network and is defined in Equation (19).

(19) Para.=numel(Θ)

where Θ denotes the network parameters, and numel(·) denotes the function used to count the number of elements in PyTorch 2.4.1.

### 4.5. Comparative Experiments and Analysis

To verify the effectiveness and comprehensive performance of the proposed model in the driver fatigue detection task, comparative experiments were conducted using a unified experimental environment and on the same dataset. Ten representative network models were selected as baselines, which could be divided into three categories. The first category comprised mainstream convolutional classification networks, including ShuffleNetV2 [28], MobileNetV2 [29], MobileNetV3 [30], EfficientNetV2 [31], and the improved MobileNetV3 [32]. The second category consisted of vision Transformer architectures, including MobileViT [33] and Swin Transformer [34]. The third category included architectures incorporating temporal or multi-feature fusion, such as the multi-stream network EMSRNet [35], the classic spatiotemporal cascade architecture InceptionV3+LSTM [36], and the spatiotemporal hybrid model CNN+LSTM [37]. The detailed experimental comparison results are shown in Table 2.

Table 2 shows that the proposed model achieved the best performance across core accuracy metrics, including accuracy (96.07%), and attained the highest inference speed (56.96 FPS), achieving an optimal balance between detection accuracy and inference efficiency. Among mainstream CNN architectures, the lightweight ShuffleNetV2 demonstrated excellent feature extraction capability (accuracy of 95.28%). Although EfficientNetV2 met accuracy requirements, its large parameter count (20.18M) led to a low frame rate (18.48 FPS), making it difficult to satisfy real-time requirements. Compared to the best-performing Improved-MobileNetV3 in this category, the proposed model achieved a 5.01% accuracy improvement with fewer parameters and more efficient inference, validating its superior network architecture. Additionally, both MobileViT and Swin-T demonstrated strong feature representation capabilities due to their self-attention mechanisms, achieving accuracies exceeding 94%. However, the self-attention mechanism inevitably introduced significant computational overhead, causing their inference speeds to hover around only 33 to 36 FPS. While maintaining extremely high accuracy (96.07%), the proposed model had less than half the parameter count of MobileViT and achieved an inference speed more than 1.6 times faster, effectively overcoming the bottleneck of Transformer architectures that struggled with high-speed inference on edge devices.

In addition, regarding architectures incorporating temporal or multi-feature fusion, it should be noted that since the original authors of EMSRNet and the classic spatiotemporal models (InceptionV3+LSTM/CNN+LSTM) did make their code open-source, rigorous code reproduction was conducted based on the original literature to ensure experimental fairness. Experiments showed that the large parameter count of the multi-stream network EMSRNet limited its operational efficiency (30.15 FPS). For spatiotemporal models incorporating LSTMs, even though the parameter count of CNN+LSTM was compressed to 1.44M, its inference speed remained only 20.78 FPS, fully exposing the serial bottleneck of traditional LSTMs in temporal computation. Moreover, the accuracy of such models only hovered around 70%, indicating that simply introducing temporal modules easily led to feature alignment difficulties and overfitting risks. In contrast, the proposed model, with a low parameter count of 2.18M and an excellent performance of 56.96 FPS, fully validated its potential for real-time fatigue detection in complex in-vehicle environments.

### 4.6. Ablation Experiments and Analysis

#### 4.6.1. Investigation of Sliding Window Size $L_{sw}$

The length of the sliding window $L_{sw}$ directly determined the temporal span of the model’s receptive field. This section tests the performance variation with window sizes $L_{sw}$ ranging from 2 to 8. The experimental results are shown in Table 3. As $L_{sw}$ increased, accuracy first rose and then fell. When $L_{sw}$ = 3, the model captured the most appropriate local temporal dependencies, achieving peak accuracy. If $L_{sw}$ continued to increase (e.g., L=8, accuracy dropped to 94.02%), this introduced excessive redundant historical information and instead interfered with the judgment of the current frame while increasing computational overhead. Therefore, we ultimately selected $L_{sw}$ = 3 as the default configuration.

#### 4.6.2. Investigation of Adaptive Feature Stream Selection Threshold τ

**Figure 7:**
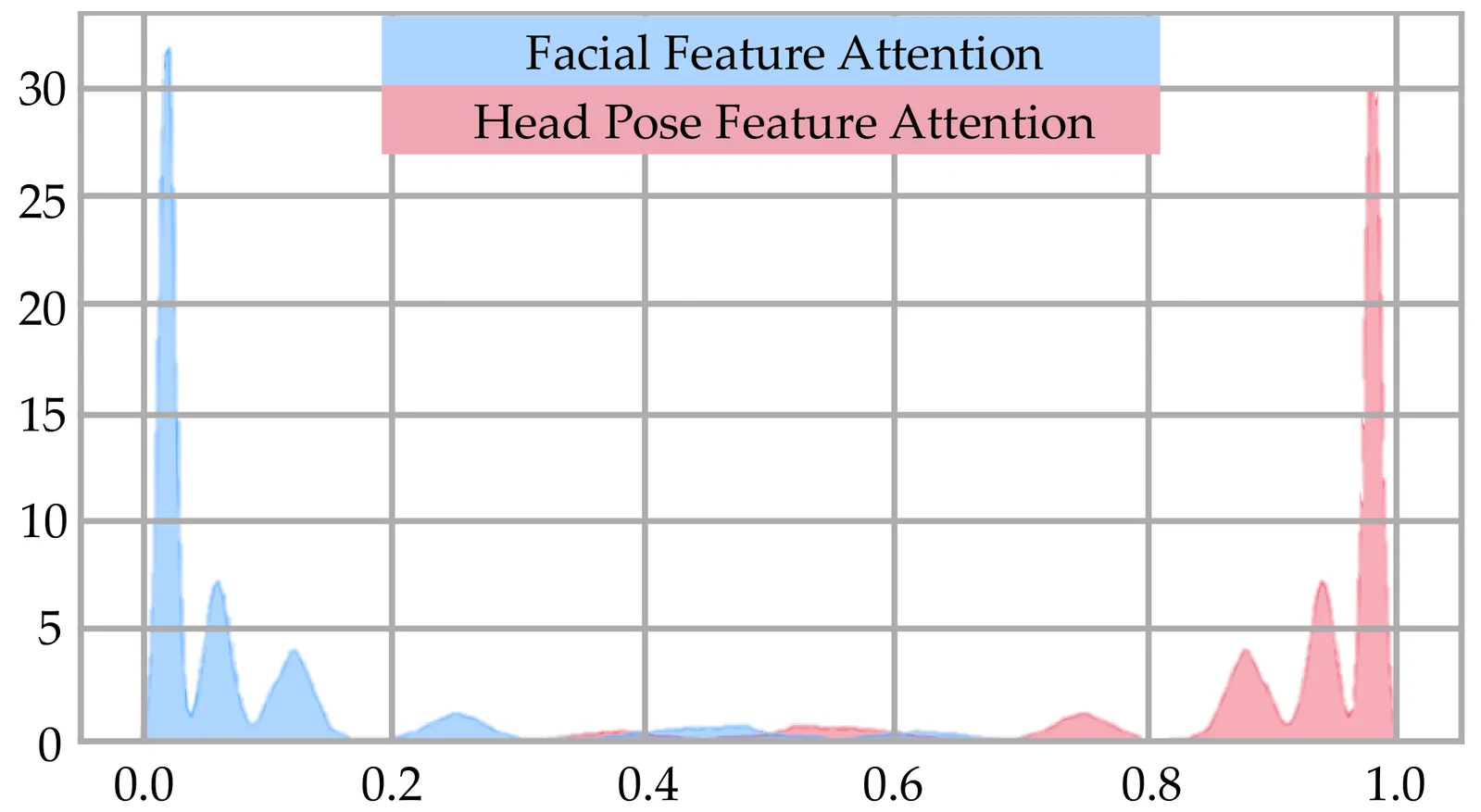
Weight density distribution of each branch in the fusion module.

Figure 7 shows the attention weight density distribution of each branch in the training stage fusion module. Due to the SigMoid normalization constraint, the sum of the weights of the facial branch (blue) and the head pose branch (red) was always 1. The distribution in the figure shows a clear polarization phenomenon, demonstrating that the model highly relied on facial features for downstream discrimination, while its dependence on head pose features was extremely low. This weight imbalance was mainly attributed to the sparsity of specific pose samples such as head lowering in the training set. This distribution pattern not only revealed the redundancy in computational resource allocation of the current dual-branch architecture but also laid an experimental foundation for the subsequent design of an adaptive inference acceleration scheme.

Table 4 records the impact of different adaptive feature stream selection thresholds τ on overall model performance. Based on the distribution pattern in Figure 7, where facial feature weights are highly concentrated above 0.82, this experiment reasonably set the threshold interval to [0.82,1.00]. The experimental results showed a trade-off between model inference speed and detection accuracy. As τ gradually increased from 0.82 to 1.00, the proportion of samples sent to the fusion branch increased from 6.7% to 100%. Although the F_1_-score of the model steadily increased from 93.67% to 95.75%, the inference speed dropped sharply from 65.13 FPS to 50.55 FPS due to the increased computational load.

In practical fatigued driving detection scenarios, the optimal threshold setting was not fixed but should be flexibly determined based on the computational resources of the specific deployment device to seek the best balance. In terms of the comprehensive performance of this experiment, it could be observed that when τ exceeded 0.96, the improvement in model accuracy and F1-score tended to slow down. In this case, we set τ=0.96 as the default hyperparameter for adaptive inference.

#### 4.6.3. Core Component Ablation Experiments

To verify the contribution of each core module to model performance, the ablation experiment results are shown in Table 5. Although the base dual-stream fusion model achieves an extremely high inference speed (66.30 FPS), its accuracy still has room for improvement (95.77%). After introducing the Temporal Module, the model can effectively capture dynamic fatigue features among video frames and achieves the highest accuracy (96.34%). However, it brings considerable computational overhead, and the frame rate drops sharply to 50.55 FPS. To alleviate this speed bottleneck, we further equip the model with the Adaptive Feature Stream Selection mechanism. This component filters redundant spatiotemporal features and recovers the inference frame rate to 56.96 FPS at the expense of a minor accuracy reduction from 96.34% to 96.07%. The overall accuracy remains obviously higher than that of the base network. These observations demonstrate that our complete architecture compensates for the speed loss introduced by temporal computation via acceptable accuracy degradation, and it obtains a favorable trade-off between detection accuracy and real-time inference.

Table 6 presents ablation studies to analyze the effects of TDF and AFS on the target dataset. The non-temporal baseline reaches an accuracy of 95.77% with an FPS of 66.30. The face-only temporal model introduces face temporal information, yielding a slight accuracy improvement (95.84%) but reducing inference speed to 61.49 FPS. Incorporating the TDF module to exploit face and local temporal features further improves accuracy to 95.96%, yet the FPS declines to 53.21 due to increased computation. To verify the advantage of the adaptive routing strategy in AFS, we construct a random routing variant. The results show that the TDF model with random routing achieves 96.07% accuracy and recovers FPS to 56.96. This observation confirms that TDF captures complementary temporal cues, and AFS adaptively distributes computation to mitigate the efficiency drop, jointly optimizing the performance–efficiency trade-off.

### 4.7. Visualization Results Analysis

#### 4.7.1. t-SNE Visualization Analysis

To intuitively verify the effectiveness of different algorithms in feature extraction and classification, we employed t-SNE [38] technology to perform dimensionality reduction visualization analysis on the features of the dual-stream network, temporal dual-stream network, and temporal feature stream selection network.

**Figure 8:**
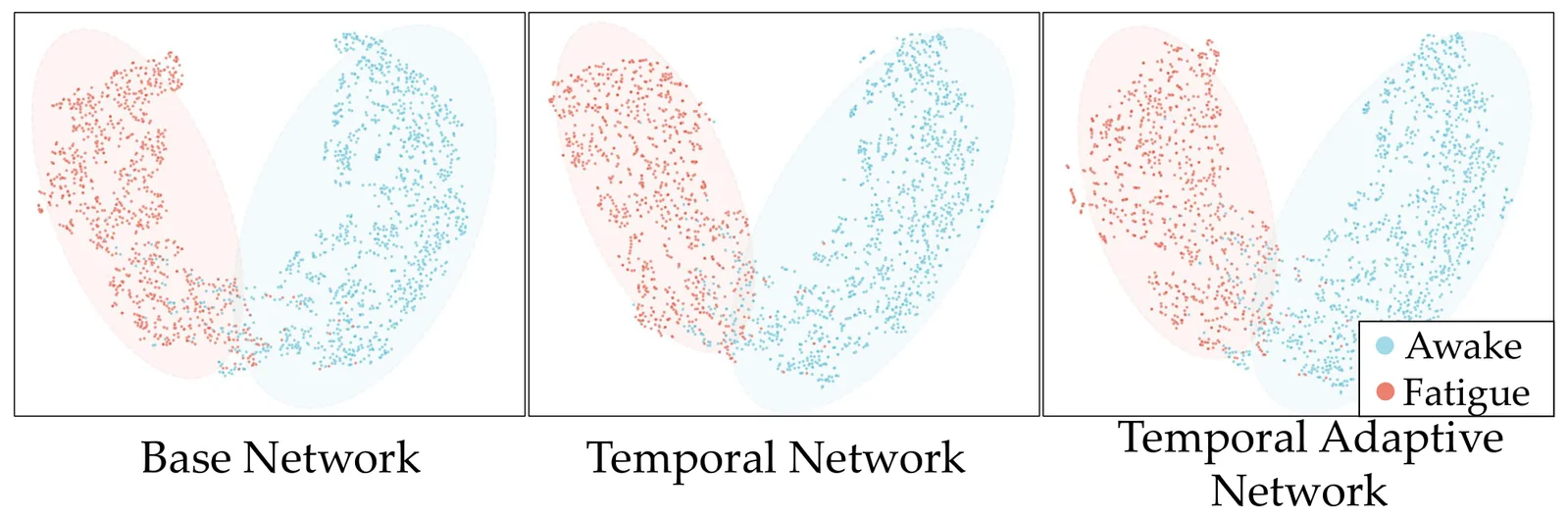
Comparison of t-SNE feature visualizations for different networks.

In Figure 8, observing the left subfigure, we saw that although the dual-stream network initially achieved separation of fatigue and alert features, the feature distribution was relatively dispersed. After introducing temporal information in the middle subfigure, the intra-class compactness of the two feature clusters was improved, forming clustering results with the highest visual separation and clarity. After further introducing the adaptive feature stream selection mechanism, the distribution of feature clusters (the right subfigure) showed a slight loosening trend compared to the middle subfigure but overall maintained good inter-class discrimination. This visual evolution process intuitively and faithfully reflected that the complete architecture proposed in this paper recovered inference speed at the cost of slightly sacrificing representation purity.

#### 4.7.2. Grad-CAM Visualization Analysis

To further investigate the focus areas of different models during feature extraction and verify the effectiveness of the proposed algorithm, we employed Grad-CAM [39] technology to perform class activation map visualization analysis on the feature extraction processes of the facial temporal network, head temporal network, and the proposed algorithm. The visualization results were superimposed on the original images in the form of heatmaps, where warmer colors indicated higher model attention to that region and greater contribution to the final prediction.

**Figure 9:**
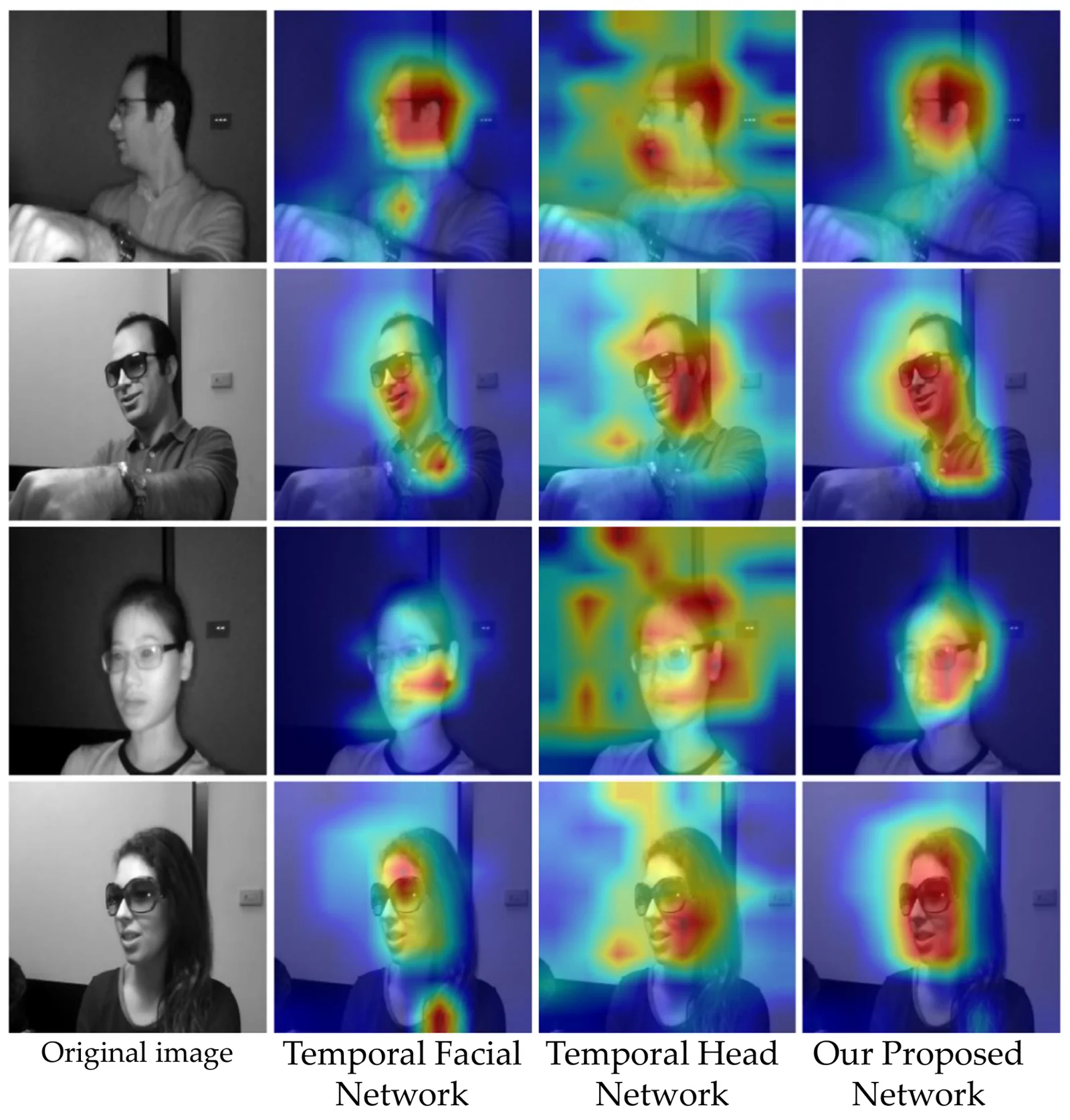
Comparison of Grad-CAM feature heatmaps for different networks.

As shown in Figure 9, by comparing four sets of samples covering complex driving scenarios such as large poses and partial occlusion, we clearly observed significant differences in feature focus areas among different networks. Compared to single-stream feature networks, the proposed network with fused dual-stream features demonstrated outstanding anti-interference capability. Specifically, facing challenging samples such as tilting, wearing glasses, and occlusion by sunglasses, the proposed network accurately and compactly anchored the high-response regions to the head area containing facial features. It not only successfully suppressed interference from irrelevant noise such as arms, torso, and background but also compensated for the limitations of single-branch networks in feature extraction. This intuitive visualization result strongly demonstrated that the proposed algorithm could fully leverage the complementary advantages of facial details and head global features, guiding the model to learn more robust and accurate fatigue discrimination features.

## 5. Conclusions

The proposed framework includes two core modules: the TDF temporal-fusion module and the AFS adaptive inference module. The TDF temporal-fusion module exploits inter-frame temporal correlations to aggregate multi-frame features, suppresses frame noise and improves fatigue detection accuracy. By contrast, the AFS adaptive inference module optimizes computational cost for embedded vehicle-side deployment. It dynamically selects inference branches to reduce redundant computation and achieves a good balance between detection accuracy and real-time performance.

Nevertheless, this method still has practical limitations for real-world scenarios. Extreme lighting degrades image quality and harms feature extraction. Heavy occlusions from hats, masks or sunglasses interfere with fatigue feature capture. Additionally, variations in camera hardware may cause domain gaps and reduce generalization performance. These problems will be explored in future work.

## Figures and Tables

**Table 1:** The details of the experimental setup.

Para. Name	Facial Net.	Head Pose Net.	Dual-Stream Net.
Input Size	3×224×224	3×64×64	3×224/64×224/64
Batch Size	32	32	32
Epochs	100	100	100
Optimizer	SGD	AdamW	SGD
Learning rate	0.01	0.001	0.001
Momentum	0.937	–	0.9
Weight decay	0.0005	0	0.0005

**Table 2:** Comparison with state-of-the-art methods on the NTHU-DDD dataset. Values are reported as percentages (%) for Acc., P, R, and F_1_, in millions (M) for Para., and in hertz (Hz) for FPS.

Method	Acc.	P	R	$F_{1}$	Para.	FPS
ShuffletV2	95.28	91.79	**97.73**	94.64	**1.26**	48.80
MobileV2	77.25	73.06	74.33	73.69	2.23	54.02
MobileV3	89.60	85.53	91.15	88.25	1.52	48.81
EfficientV2	91.35	87.91	92.56	90.17	20.18	18.48
Improved-MobileV3	91.06	91.38	90.96	91.02	2.69	50.37
MobileViT	95.82	**95.78**	94.39	95.08	4.94	33.75
Swin-T	94.54	94.39	94.48	94.44	27.50	36.57
EMSRNet	76.65	83.59	76.65	76.29	26.08	30.15
InceptionV3+LSTM	70.21	69.05	65.33	65.73	10.40	11.28
CNN+LSTM	72.45	64.71	83.62	76.52	1.44	20.78
**Ours**	**96.07**	94.98	95.90	**95.44**	2.18	**56.96**

**Table 3:** Hyperparameter study of length of sliding window $L_{sw}$.

$L_{sw}$	Acc.(%)	P(%)	R(%)	$F_{1}$(%)	Para.(M)	FPS
2	95.80	94.62	95.63	95.12	2.18	58.12
3	**96.07**	**94.98**	**95.90**	**95.44**	2.18	56.96
4	95.56	94.31	95.34	94.82	2.18	48.01
5	95.19	93.92	94.93	94.42	2.18	44.70
6	94.30	92.86	93.96	93.41	2.18	42.53
7	94.15	92.64	93.74	93.19	2.18	40.21
8	94.02	92.54	93.64	93.09	2.18	38.15

**Table 4:** Ablation study on the adaptive feature stream selection threshold τ (% except for FPS).

τ	Acc.	P	R	F_1_	FPS	Ratio
0.82	94.54	93.07	94.28	93.67	65.13	92.8∼0.50∼6.70
0.92	95.13	93.81	94.92	94.36	62.19	76.8∼0.20∼23.0
0.94	95.62	94.34	95.46	94.89	59.37	63.4∼0.10∼36.5
0.96	96.07	94.98	95.90	95.44	56.96	48.9∼0.00∼51.1
0.98	96.11	95.08	95.90	95.49	53.21	21.3∼0.00∼78.7
1.00	**96.34**	**95.29**	**96.22**	**95.75**	50.55	0.00∼0.00∼100

**Table 5:** Ablation study of the core components using five different random seeds.

Temporal	F-Selection	Acc.	P	R	F_1_	FPS
		95.77 ± 0.31	94.36	95.29	94.51	66.30
✓		96.34 ± 0.28	95.29	96.22	95.75	50.55
✓	✓	96.07 ± 0.34	94.98	95.90	95.44	56.96

**Table 6:** Ablation study of TDF and AFS on the target dataset.

Model	Temporal Input	Routing	Acc (%)	FPS
Non-temporal baseline	None	–	95.77	66.30
Face-only temporal	Face	Adaptive	95.84	61.49
+ TDF	Face + Local	Adaptive	95.96	53.21
+ TDF+AFS	Face + Local	Random	96.07	56.96

## Data Availability

The NTHU Driver Drowsiness Detection (NTHU-DDD) dataset used in this study is publicly available at: http://cv.cs.nthu.edu.tw/php/callforpaper/datasets/DDD/, (accessed on 1 September 2026).

## Abbreviations

The following abbreviations are used in this manuscript:

AFS	Adaptive feature selection
DMS	Driver Monitoring System
DSAF	Dual-Stream Attention Fusion
FPS	Frames Per Second
GAP	Global Average Pooling
GRU	Gated Recurrent Unit
NTHU-DDD	NTHU Driver Drowsiness Detection
TDF	Temporal Dual-Stream Fusion
